# Wired to Connect: The Autonomic Socioemotional Reflex Arc

**DOI:** 10.3389/fpsyg.2022.841207

**Published:** 2022-06-24

**Authors:** Robert J. Ludwig, Martha G. Welch

**Affiliations:** ^1^Department of Pediatrics, Columbia University Irving Medical Center, New York, NY, United States; ^2^Department of Psychiatry, Columbia University Irving Medical Center, New York, NY, United States; ^3^Department of Anatomy and Cell Biology, Columbia University Irving Medical Center, New York, NY, United States

**Keywords:** approach-avoidance, state–trait, brainstem, signaling pathway, emotion, instinct, prematurity, mother–child

## Abstract

We have previously proposed that mothers and infants co-regulate one another’s autonomic state through an autonomic conditioning mechanism, which starts during gestation and results in the formation of autonomic socioemotional reflexes (ASRs) following birth. Theoretically, autonomic physiology associated with the ASR should correlate concomitantly with behaviors of mother and infant, although the neuronal pathway by which this phenomenon occurs has not been elucidated. In this paper, we consider the neuronal pathway by which sensory stimuli between a mother and her baby/child affect the physiology and emotional behavior of each. We divide our paper into two parts. In the **first part**, to gain perspective on current theories on the subject, we conduct a 500-year narrative history of scientific investigations into the human nervous system and theories that describe the neuronal pathway between sensory stimulus and emotional behavior. We then review inconsistencies between several currently accepted theories and recent data. In the **second part,** we lay out a new theory of emotions that describes how sensory stimuli between mother and baby unconsciously control the behavior and physiology of both. We present a theory of mother/infant emotion based on a set of assumptions fundamentally different from current theories. Briefly, we propose that mother/infant sensory stimuli trigger conditional autonomic socioemotional reflexes (ASRs), which drive cardiac function and behavior without the benefit of the thalamus, amygdala or cortex. We hold that the ASR is shaped by an evolutionarily conserved autonomic learning mechanism (i.e., functional Pavlovian conditioning) that forms between mother and fetus during gestation and continues following birth. We highlight our own and others research findings over the past 15 years that support our contention that mother/infant socioemotional behavior is driven by mutual autonomic *state* plasticity, as opposed to cortical *trait* plasticity. We review a novel assessment tool designed to measure the behaviors associated with the ASR phenomenon. Finally, we discuss the significance of our theory for the treatment of mothers and infants with socioemotional disorders.

## Introduction

Our clinical observations, combined with our basic and clinical research, support a new physiological and behavioral construct we have labeled *autonomic emotional connection*. We have proposed that mothers and infants co-regulate one another’s autonomic state through an autonomic conditioning process, which starts during gestation and results in the formation of an interpersonal autonomic socioemotional reflexes (ASRs; Ludwig and Welch, 2020). Our clinical research findings among mothers and preterm infants in the neonatal intensive care unit (NICU) have demonstrated the efficacy of an intervention aimed at emotional connection and autonomic co-regulation as a basis of optimal development (Welch and Myers, 2016; Welch et al., 2020). These co-learned conditional reflexes drive measurable mother/infant socioemotional behaviors after birth. We labeled this special class of reflex the *autonomic socioemotional reflex* (*ASR*).

We have previously reviewed two theoretical questions that Darwin struggled to answer: What is the role of conscious vs. unconscious control of heart rate in emotional behavior? And, what is origin and function of mother/infant instinctive behaviors? (Ludwig and Welch, 2019). Darwin concluded that emotions as well as heart rate are controlled consciously. Regarding mother-infant instinctive behaviors, Darwin concluded: (1) they are inherited and (2) they should theoretically be expunged through evolution. We argued that although Darwin clearly stated his conclusions were largely hypothetical, both his theories and his assumptions on mother-infant emotional behavior have become scientific dogma.

In this paper, we challenge Darwin’s idea that so-called instinctive or innate mother/infant emotional behaviors and heart rates are inherited and self-regulated. We conduct a narrative review of reflex arc theory, showing its progression over 500 years to currently accepted theory on cardiac reflexes associated with emotional behavior. We review several accepted theories on emotions, which include top-down, self-control of cardiac function and emotional behavior *via* an internal signaling pathway. We then propose a novel theory of emotions, which states that mother/infant socioemotional behavior and autonomic function are controlled externally *via* mutual brainstem reflex signaling mechanisms. We describe the ASR signaling arc, which begins with mother-infant sensory stimuli and ends with emotional behavior and autonomic state. Finally, we review evidence supporting our theory of emotion and discuss its clinical implications for infant development, mother/infant relational health, and the prevention and treatment of maladaptive socioemotional behaviors and dysregulated physiology of each and between mother and infant/child.

## Historical Review—The Reflex Arc and Emotions

After the teachings of Greek physician, surgeon and philosopher Galen (129–204 CE), there was virtually no advancement in the knowledge of the structure and functions of the living organism for more than a thousand years (Fearing, 1964). Then, in the sixteenth Century Belgian anatomist and physician, Andreas Vesalius (1514–1564), figuratively *broke the doors of Galenic doctrinal authority down* and ushered in a new scientific era of verifying theory through direct rigorous experiment. Once opened, advances in biological science were relatively rapid and dramatic. While Vesalius was primarily interested in the structure of neural action, scientists quickly turned their attention to the nature of neural action and nervous control of behavior. These new discoveries in physiology pitted the scientific method against various religious beliefs and dogmas. Over the next two centuries, a flood of biological discoveries promoted new theories on the nature of human behavior. In 1628, Harvey (1578–1667) published evidence that bodily action is a physical phenomenon governed in part by circulating blood. The work of Dutch naturalist Jan Swannerdam (1606–1680), and physiologists Hermann Boerhaave (1668–1738), Albrecht von Haller (1708–1777) and Robert Whytt (1714–1766) began to reveal the mysteries of voluntary and involuntary muscular action, and how each is controlled.

**Figure 1:**
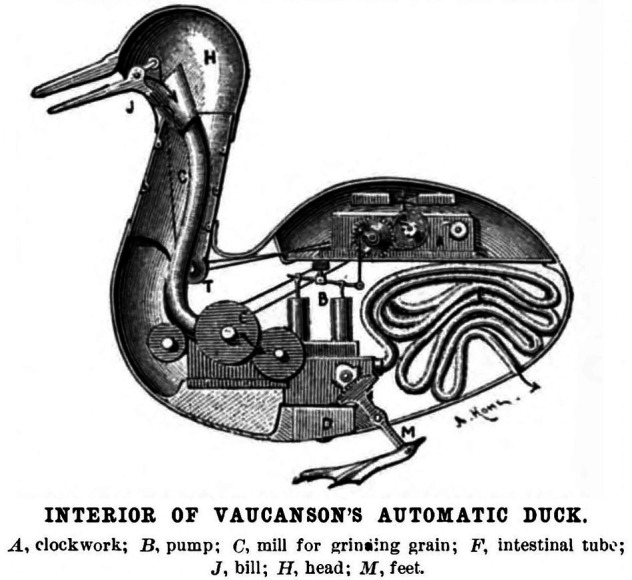
**Schematic of Vaucanson’s Canard Digérateur, or Digesting Duck**. Inspired by Descartes’ idea that animals function much like a machine, Vaucanson created and unveiled a working model of the duck automaton pictured above in 1739. Two hundred years later in the 1940’s, Norbert Wiener theorized that all intelligent behavior functions as result of feedback mechanisms, much like a machine. The idea that humans function like machines has not changed for 400 years. It remains today a core assumption of neuroscience and artificial intelligence. (Image public domain). Source: Scientific American (1899).

In 1649, French philosopher and scientist René Descartes proposed a mechanism by which the body’s perceived impulses lead to action. Descartes’ neuronal pathway came to be known as a *reflex arc*. According to Descartes, mind and body processes are separate. The actions of the body are simple unconscious actions, much like a machine (Figure 1).

Actions of the mind on the other hand are meaningful, conscious, and voluntary. Descartes’ reflex arc model described sensory input and motor output managed by the brain. For Descartes this concept was existential: *I think*, therefore *I am*. Descartes proposed that our mind makes us distinctly *human* and separates us from our animal instincts and impulses (Decartes, 2008). The idea resonated with the Judeo-Christian religious belief that God “hast made him [man] a little lower than the angels, and hast crowned him with glory and honor” (Psalm-8.5 Bible, n.d.).

Throughout most of the eighteenth century, the Cartesian dualistic relationship between nerve structure and function was still the organizing principle for the study of emotional behavior. No matter what psychological processes may occur concomitantly, researchers believed the body’s nervous system is a series of simple reflexes. By the end of the eighteenth century, however, the dualistic relationship between mind and function had given way to the concept that nerve function is not separate from thought processes. By 1900, the modern definition of reflex arc was defined as *a stimulus and a response*. This definition remains approximately the same today.

Tensions between religion and science that boiled over in the sixteenth century gradually eased in the seventeenth and eighteenth centuries, as teaching within both spheres generally aligned on the earth’s place and movement in the heavenly bodies and man’s singular hierarchical place in the animal kingdom. By the middle of the nineteenth century, however, a new quest to deepen the understanding of fundamental principles of physics and chemistry emerged. At the same time, a similar quest emerged within physiology and psychology to understand the fundamental principles of the reflex arc as they pertained to emotional behavior (Fearing, 1964). Thus, the psychophysiology debate shifted from metaphysical mind–body dualism involving constructs such as *soul, animal spirits, and nervous fluids* to a scientific brain–body debate over *reflex action, mechanism and control of behavior*.

English naturalist and biologist Charles Darwin (1809–1882) set the stage for the modern debate over the reflex arc with his theories on emotional behavior (Darwin, 1872). He spent the latter part of his career pondering the separate effects of cardiac reflexes and the brain on instinctive behavior and emotions. On the origin of emotional behavior, Darwin accepted that emotional behavior arises from reflex actions in the body (i.e., bodily impulses). Summarizing the accumulated knowledge about reflexes at the time, Darwin wrote:

“*Reflex actions, in the strict sense of the term, are due to the excitement of a peripheral nerve, which transmits its influence to certain nerve cells, and these in their turn excite certain muscles or glands into action; and all this may take place without any sensation or consciousness on our part…*” (Darwin, 1872).

Darwin drew special notice to the teachings of the French physiologist Claude Bernard, who repeatedly insisted that the sequence of events ending in emotional behavior begins with action on the heart.

“*…when the heart is affected it reacts on the brain; and the state of the brain again reacts through the pneumo-gastric [vagus] nerve on the heart; so that under any excitement there will be much mutual action and reaction between these, the two most important organs of the body*” (Darwin, 1872).

If the heart can influence the brain, Darwin wondered, what is the role of unconscious reflex mechanisms in evolution?

“*When movements, associated through habit with certain states of the mind, are partially repressed by the will, the strictly involuntary muscles, as well as those which are least under the separate control of the will, are liable still to act*” (Darwin, 1872).

In the end, Darwin could not answer with any certainty the role of conscious vs. unconscious control of heart rate in emotional behavior. He concluded his many years of thinking on the brain/heart subject with a call to future researchers.

“*From these several causes, we may conclude that the philosophy of our subject [*i.e.*, the expression of emotions in man and animals] has well deserved the attention which it has already received from several excellent observers, and that it deserves still further attention, especially from any able physiologist*” (Darwin, 1872).

European and Russian neurologists, physiologists and psychologists quickly took up Darwin’s call, establishing a new field of psychophysiology. The concept that the entire nervous system works on a single reflex pattern dominated the work of Russian psycho-physiologists Ivan Sechenov (1829–1905), Ivan Pavlov (1849–1936) and English neurophysiologist Charles Scott Sherrington (1957–1952), as well as Austrian neurologist Sigmund Freud (1856–1939; Amacher, 1964). A consensus emerged that a sensory stimulus initiates a nervous impulse in a peripheral nerve. The impulse passes through nervous centers and from there in turn passes to peripheral motor nerves. By the turn of the twentieth century, Descartes’ idea that sensory stimulus, central connections and motor responses were separate and complete entities in themselves had given way to a new idea, which was described in 1896 by American philosopher and psychologist Dewey (1896). Dewey included the same components of the reflex arc, but he now described them as divisions of labor and functioning factors as a single concrete whole within the self (Phillips, 1971). This view of the reflex arc has essentially not changed since Dewey introduced it.

Today, a reflex arc is defined as a neural pathway that controls a reflex action (Saladin, 2015). Broadly speaking, a reflex arc is thought of as a functional system that allows the human body to adapt to its external environment and to its internal milieu (Conley et al., 2017). The major neuron types include sensory and motor neurons and interneurons. Interneurons (also known as association neurons) are neurons that are found in the CNS (brain and spinal cord), as well as in the enteric nervous system (Furness et al., 2014). They connect to other interneurons, allowing them to communicate with one another. Thus, reflex arcs have interneurons that act as “middle-men” between *sensory* (afferent) neurons and *motor* (efferent) neurons.

In vertebrates, few sensory impulses pass directly through the cortex and reach consciousness. Instead, most sensory neurons synapse in the brainstem. This allows faster reflex actions to occur by activating motor neurons without the delay of routing signals through the cortex. The cortex processes the sensory input *via* interneurons while the reflex leads to motor action. In this way, analysis of the signal takes place within the cortex after the reflex motor action has taken place. With few exceptions, autonomic reflex arcs project from the spinal cord or brainstem and synapse on the ganglionic neurons that project to effectors in the viscera. They affect inner organs, such as the heart, and serve to either disrupt or maintain homeostasis (Yuste, 2005).

Autonomic reflex arcs connect sensory neurons to motor neurons without first reaching the cortex. A broad range of autonomic reflexes control blood pressure, breathing, ensure tissue oxygenation, and otherwise act in concert to maintain homeostasis (Conley et al., 2017). After an autonomic reflex action, the ANS conveys impulse signals from the vasculature, the heart, and other internal organs, primarily *via* the vagus nerve to the central nervous system (e.g., medulla, pons and hypothalamus). Various autonomic reflexes, such as the *orienting reflex*, have been studied to understand control of heart rate and the brain’s role in regulating emotional responses.

### The Orienting Response

An organism’s immediate response to novel or significant stimuli in its environment was first described by Sechenov in his 1863 book *Reflexes of the Brain*. Sechenov’s student, Ivan Pavlov, termed the immediate reflex action the *orienting reflex* (Pavlov, 1927). The orienting reflex phenomenon has become an important area of research on the emotional pathologies in infants and children (e.g., fear, anxiety, depression), since orienting and attention stem from activation of highly conserved defensive and appetitive motivational systems that evolved to protect and sustain life (Porges, 2011). Orienting is considered the leading edge of the brain’s guidance of behavior. As such, it provides a spatial framework upon which the more complex functions of perception, discrimination, recognition, and visual motor responses operate effectively (Sprague, 1996).

At first, researchers concentrated on directional behavioral movements of head and eye related to the orienting reflex. But, with advances in physiological assessment emerging after World War II, researchers, such as the Russian psycho-physiologist Evgeny Sokolov (1920–2008), began finding multiple autonomic phenomena associated with the orienting reflex, including changes in phasic and galvanic skin response (GSR), electroencephalogram (EEG), and heart rate after a novel or significant stimulus. Sokolov found the immediate orienting effects occurred in less than 1 s of introducing the stimulus (Sokolov, 1960).

In addition to the immediate effects, however, Sokolov documented a phenomenon related to the orienting reflex he called “habituation,” referring to a gradual “familiarity effect” and reduction of the orienting response with repeated stimulus presentations. In addition to the immediate autonomic effects, Sokolov widened his investigation to study the longer cortical *after effects* of the reflex, what he called the orienting “*response*” (e.g., how the organism behaves after the immediate orienting autonomic reflex or after repeated stimuli). Unfortunately, the same OR abbreviation is frequently used to refer to the two phenomena interchangeably, thus blurring the distinction between the reflex action and the response action. To avoid this confusion, we will henceforth refer to the cardiac ANS part of the phenomenon as the *orienting REFLEX* and the cortical *CNS* part of the phenomenon as the later orienting *RESPONSE*.

Sokolov and other psycho-physiologists systematically investigated the orienting RESPONSE in relationship to cardiac mechanisms controlling heart rate and emotional behavior, specifically *heart rate deceleration*, which is mediated by the parasympathetic branch of the ANS (Lang et al., 1997). Orienting leads to a rapid, brief deceleration of heart rate to any novel stimulus. Until the late eighteenth century it was believed that all branches of the sympathetic and parasympathetic branches of the ANS responded *via* reciprocal innervation. That is, if one set of muscles receive a signal for a reflex action, the antagonistic set of receives a simultaneous signal that inhibits action. However it is now understood that the two systems can be co-active (Latash, 2018), depending on the stimulus context (Berntson et al., 1994). Co-activation is clearly seen during orienting, which involves both motor inhibition and heart rate deceleration (Bradley et al., 2012).

By the late 1960s and 1970s, the theoretical consensus among psycho-physiologists was that orienting initiates a cascade of perceptual unconscious and conscious input and motor output that prepares the organism to react and facilitates the selection of appropriate behavior. Thus, their sequence of the orienting phenomenon included, in order: (1) determination of *significance*; (2) cortical processing; (3) initial cardiac deceleration; (4) preparation for action; and finally, (5) cardiac and electrodermal response. In other words, *the organism must think before it acts*. In their view, orienting (i.e., action) comes only after cortical processing.

However, by the 1980’s anomalies began to appear in experimental data testing the theory. Both the *novelty* of the stimulus and the *significance* of the stimulus appeared to alter results. In addition, different components of the orienting REFLEX habituated at different rates. Specifically, some evidence showed that an orienting RESPONSE sometimes does not occur, despite changing the stimulus. In other cases, some individuals showed a RESPONSE and others did not. In addition, it was clear that instructions given to the subjects and the relevance of the task involved could affect both the magnitude of the response and the rate of habituation. Thus, both *novelty* and *significance of the stimulus* can affect the magnitude and probability of the orienting RESPONSE. Apparently, as clinical psychologist Margaret M. Bradley (1955) summed up the predicament of behavioral reflex research at the time, it was becoming clear that “not all changes in stimulus are equal” (Bradley, 2009).

**Figure 2:**
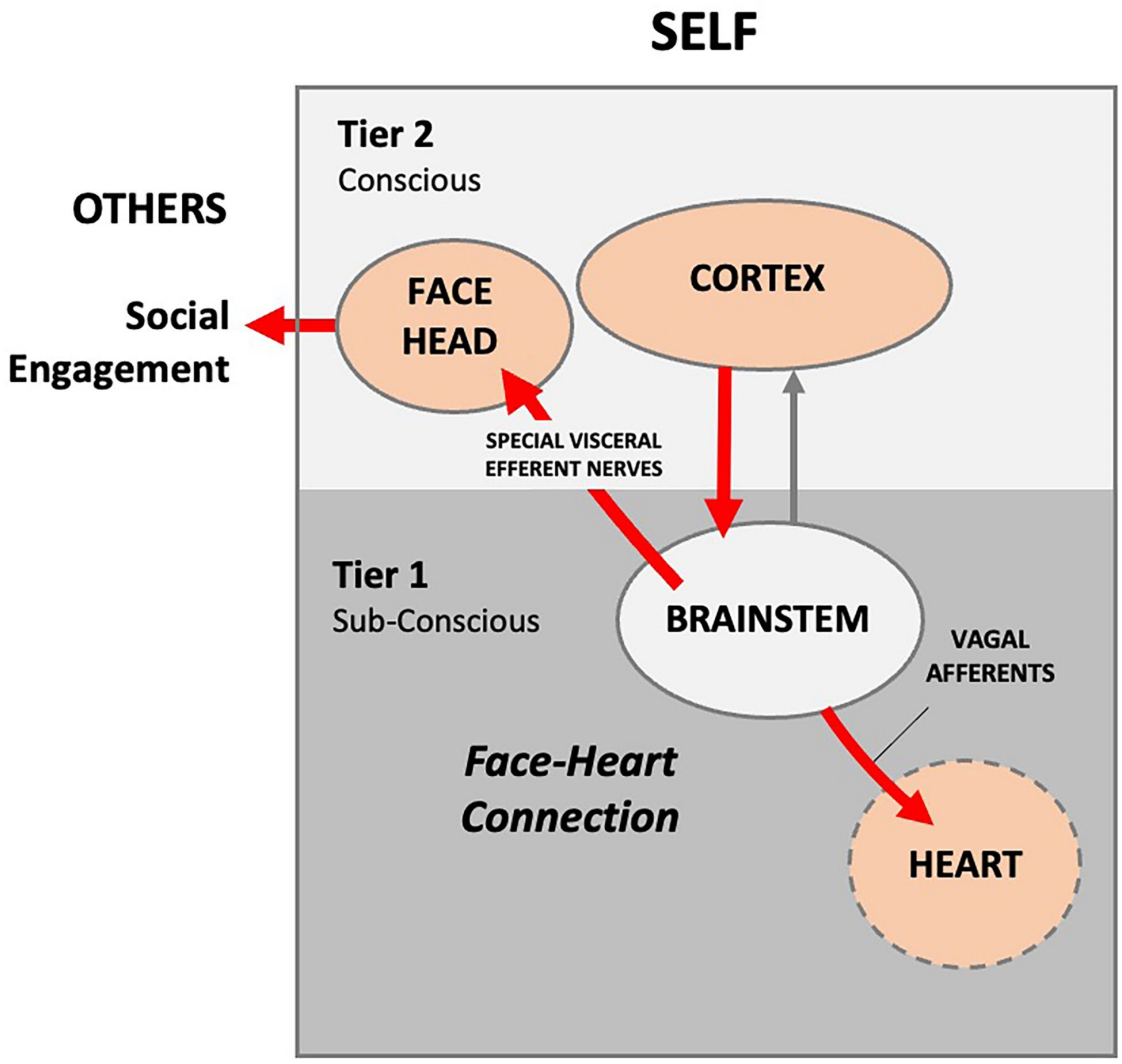
**Author’s conceptualization of the Polyvagal Social Engagement System**. Internal signals are routed to the cortex, where the information is processed. The cortex sends conscious voluntary commands to the brainstem and thence to the facial muscles and heart *via* cranial nerves (red lines), thus creating a theoretical face-heart connection. In this model, social engagement is entirely outward facing. External social signaling is not accounted for and the signaling arc is entirely internal. Presumably, the infant and mother each consciously self-regulate their autonomic functioning *via* facial and head muscles.

In 1995, Stephen W. Porges (1945) published *Orienting in a defensive world: Mammalian modifications of our evolutionary heritage. A Polyvagal Theory* (Porges, 1995). Porges sought to provide an explanation for unresolved anomalies in the measurement of cortical control of cardiac function during social orienting. Porges proposed that disparities in cardiac function data, which he labeled the *vagal paradox*, can be explained by identifying the relationship between visceral experiences and parasympathetic vagal control of the heart. The theory proposes that mammals possess two vagal systems or pathways. One is a conserved amphibian/reptilian orienting pathway that evolved to control vegetative functions. Porges averred that, like the appendix, the reptilian REFLEX pathway is a relic left over from older phyla. The reptilian REFLEX pathway controls heart rate unconsciously *via* slower unmyelinated vagal nerves that synapse in the medulla’s dorsal motor nucleus of the vagus (DMV). A second higher-order RESPONSE pathway evolved in mammals to provide conscious and voluntary control of social orienting *via* the NTS and NA nuclei in the medulla. In the newer pathway, autonomic state provides a neural platform for social behavior (Porges and Furman, 2011). Sensory signals transmit to the cortex *via* interneurons, where the conscious brain provides functional executive modulation of heart rate *via* facial muscles and myelinated vagal efferent nerves (Porges et al., 1996). A *face-heart* connection evolved in mammals as source nuclei of vagal pathways shifted ventrally from the phylogenetically older dorsal motor nucleus (e.g., unmyelinated vagal pathways) to the nucleus ambiguus (e.g., myelinated vagal pathways). This resulted in an anatomical and neurophysiological linkage between neural regulation of the heart *via* the myelinated vagus and the special visceral efferent pathways that regulate the striated muscles of the face, head, and neck. Together this linkage between brainstem motor systems responsible for cardiovascular functions and those necessary for regulating the face, head, and neck form what Porges terms an integrated “*Social Engagement System*” (Figure 2).

Porges further posits that the older and newer vagal systems use different response strategies to overcome social challenge and stress associated with orienting. Anomalies in the vagal data can be explained by the fact that the two strategies sometimes respond in a paradoxical manner, perhaps because environmental conditions are different, or because some individuals may be better than others at self-regulating their emotions.

Polyvagal theory is based upon Norbert Weiner’s control theory (Weiner, 1948), which echoes what Descartes’ maintained—in terms of functionality, humans are essentially *machines*. Weiner argued that human physiological homeostasis is controlled by way of information feedback. In the Polyvagal model, the cortex processes the internal and external signals (e.g., information) and sends signals to facial muscles, which then control the emotional RESPONSE (e.g., facial and vocal expression, social gesture and orientation; Porges, 2011). Collectively, these muscles self-regulate social engagement by modulating the sensory features of the environment. One advantage of the cortical pathway, Porges argues, is that heart rate is controlled *via* a faster myelinated vagal nerve pathway, as opposed to the archaic reptilian REFLEX arc, which involves slower unmyelinated vagal nerves.

**Figure 3:**
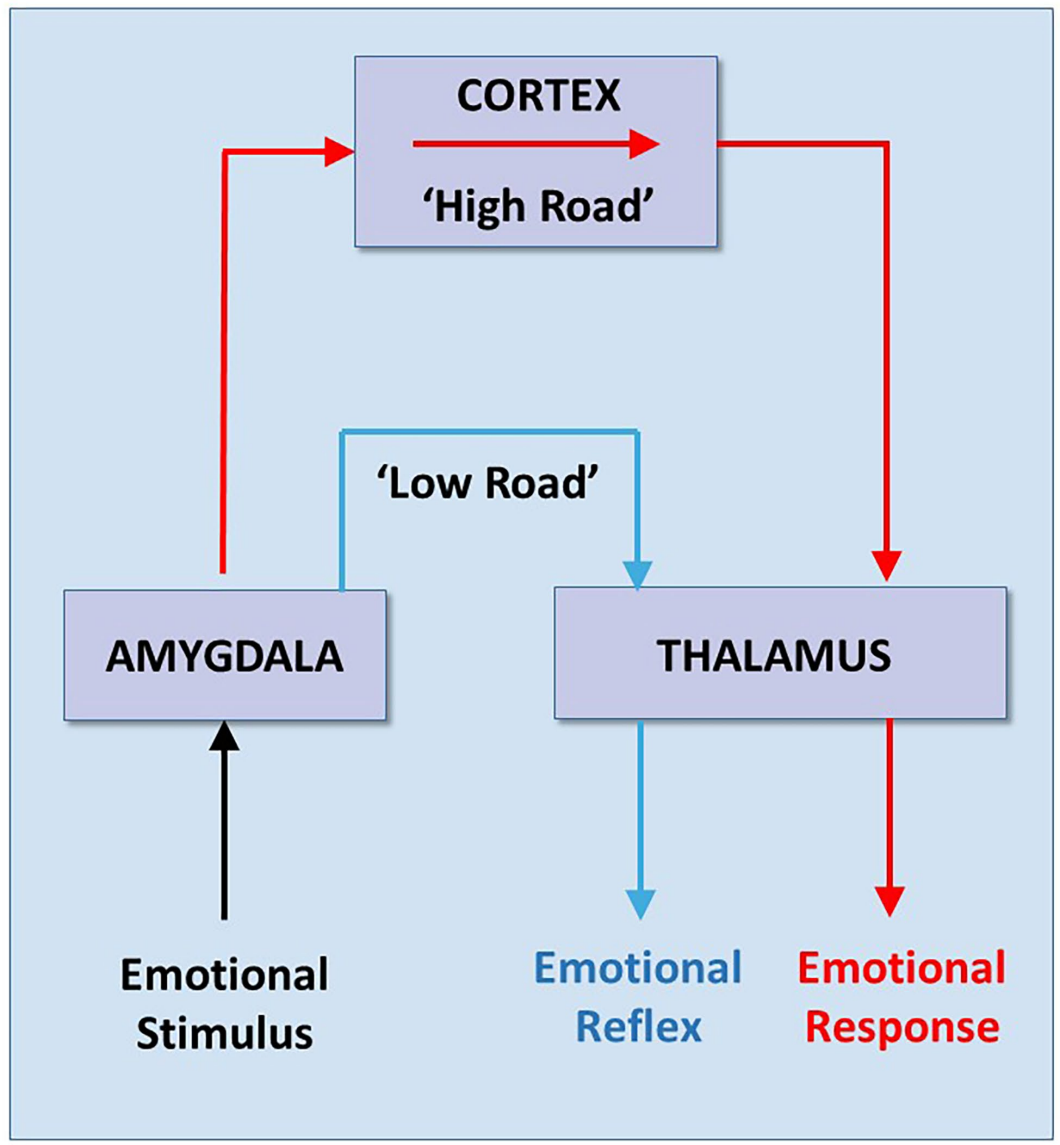
**Low and High Road reactions to Fear**. Based on experiments in rats, Joseph LeDoux developed his theory that there are two signaling pathways that generate two different emotional reactions—a “lower” and faster but crude subcortical reaction (e.g., REFLEX), (blue line), and a “higher” and slower but more accurate cortical reaction (e.g., RESPONSE) (red line). Note that both reactions begin as an emotional “stimulus” from the body, but the stimulus pathway is not described. Adapted with permission from LeDoux (1996).

*About the same time Porges was emphasizing the advantage of speed in the mammalian cortical pathway nerves*, Joseph E. LeDoux (1949) was *emphasizing the advantage of slowness in the mammalian cortical pathway*, arguing that it was more reliable in the self-regulation of emotions. In 1996, LeDoux published his influential cognitive theory on “survival circuitry.” LeDoux described a theoretical signaling pathway between an environmental stimulus and emotional response, primarily fear and anxiety (LeDoux, 1996). Based on his and others’ experiments in rats, LeDoux proposed that fear is processed *via* two different pathways in the brain. Each pathway starts with an emotional stimulus that arises from primary sensory centers in the brainstem. One *fast* pathway transmits the emotional impulses subconsciously through the *lower order* thalamus directly to the amygdala, where they are processed quickly. This pathway results in a *primitive* behavioral REFLEX. A second conscious pathway routes the sensory impulse to the higher order cortex, where it is processed *more slowly* and results in a more *accurate and informed* RESPONSE (Figure 3).

“*Information about external stimuli reaches the amygdala by way of direct pathways from the thalamus (**the low road**) as well as by way of pathways from the thalamus to the cortex to the amygdala (**the high road**). The direct thalamo-amygdala path is a shorter and thus a **faster route** than the pathway from the thalamus through the cortex to the amygdala. However, because the direct pathway bypasses the cortex, it is unable to benefit from cortical processing. As a result, it can only provide the amygdala with a crude representation of the stimulus. It is thus a quick and dirty processing pathway. The direct pathway allows us to begin to respond to potentially dangerous stimuli before we fully know what the stimulus is. This can be very useful in dangerous situations. However, its utility requires that the cortical pathway is responsible for the control of emotional responses that we do not understand. This may occur in all of us some of the time, but may be a predominant mode of functioning in individuals with certain emotional disorders*” (LeDoux, 1996).

LeDoux argued that the amygdala may release hormones due to a trigger (such as some animals have an inborn REFLEX reaction to seeing a snake). However, the sensory input is processed through cognitive and conscious processes. He identifies a primitive *defense* system that evolved over time, pointing out that even simple organisms such as bacteria have an automatic REFLEX to threats. The defense system is distinct from a higher order emotional RESPONSE system in humans, which is characterized by behaviors, such as fear and anxiety. Defensive survival circuits are theorized to exist to detect and react quickly to threats and can be present in all organisms. However, LeDoux hypothesizes that only organisms that are conscious of their own brain’s activities, such as humans, can feel fear.

LeDoux seems to echo Decartes in his view of lower order bodily mechanisms and the purer “mind.” His view of conscious RESPONSE vs. unconscious REFLEX when it comes to emotional behavior is hierarchical:

“*Breathing and believing are pretty distinct functions, clearly mediated by different brain regions. Breathing is controlled in the medulla oblongata, that utility station in the subbasement of the brain, whereas believing, like all good higher cognitive functions, goes on up the neocortical penthouse. Contrasting these is not so interesting*” (LeDoux, 1996).

### The Orienting Reflex

Research on signaling pathways and mechanisms in the brainstem over the past 20 years is challenging theoretical models focused on the conscious cortical orienting RESPONSE pathway, especially with regard to the length of time there is between sensory stimulus and a given behavioral action.

Prior to the mid-1800s scientists did not consider the length of time electrical signals take to transit a neuron because they believed the speeds were too fast to measure. In 1849, German physicist and physician Hermann von Helmholtz (1821–1894) used the newly invented galvanometer to measure how long it takes a signal to transmit through the sciatic nerve and calf muscle of a frog. He recorded transmission speeds in the range of 25–38 m/s (Glynn, 2010). Soon after, in 1862, Ivan Sechenov demonstrated that brain activity is linked to electric currents, prompting the technological revolution in electroencephalography (EEG) techniques.

In the twentieth century, physiologists began measuring multiple modes of stimuli, such as auditory and visual reflexes in response to light or sound signals. They found that factors like intensity and duration of the stimulus, age and gender of the participant and practice affected the neuronal transmission time and thus the reaction time of an individual to any particular stimulus. For instance, in 1973, psychologist Kemp (1973) showed that the time it takes an auditory stimulus to reach the brain is faster (8–10 ms) than a visual stimulus (20–40 ms), implying that we react to auditory signals faster than to visual signals. This was confirmed in 2010 by Shelton and Kumar, who showed auditory reaction times (~250 ms) are faster than the visual reaction times (~330 ms; Shelton and Kumar, 2010).

By the end of the twentieth century, measuring reaction times was a primary part of orienting research, since reacting quickly to a stimulus is a matter of life or death for nearly all species (Korn and Faber, 1996). Neurobiologists had developed new measuring techniques, such as transneuronal labeling of functionally related circuits of neurons, *in vivo* imaging of activity-related cell populations and genetic manipulations that can be used to study reaction times related to orienting escape behaviors in species such as zebrafish. French neuroscientist Henri Korn (1934) and others demonstrated that the neural circuits mediating escape reactions in both mammals and lower vertebrates have *a common single cell REFLEX arc framework* (Korn and Faber, 1996, 2005). Escape behavior has priority over ongoing behaviors (e.g., delayed cortical RESPONSES), and this direct linkage from sense organs in the brainstem to muscles ensures the fastest possible reaction time.

### Orienting and the Brainstem

Over the past two decades, the ability of ethologists to study the neurobiology of social behaviors of insects, fish, reptiles and mammals in their natural environment has grown exponentially. Increasingly smaller and more sophisticated technology has allowed researchers to observe and assess signaling pathways and reaction times at the level of milliseconds. These technological advances have opened windows on how an organism first perceives and processes novel environmental signals. This has led to revelations on the developmental roots and mechanisms of human socioemotional behavior.

**Figure 4:**
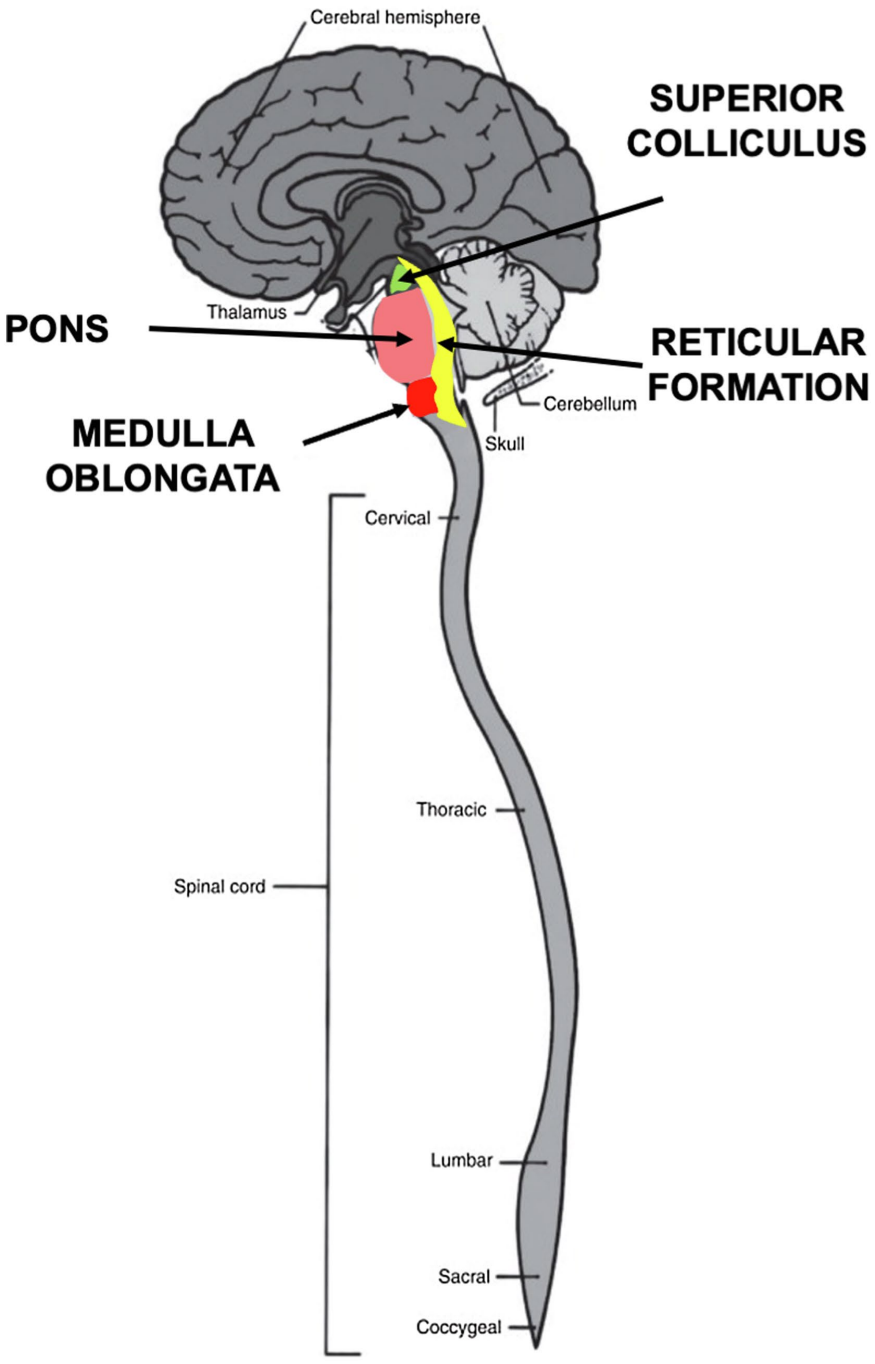
**Brainstem nuclei involved in orienting behaviors**. Four nuclei in the brainstem process sensory stimuli during orienting. The superior colliculus in the midbrain integrates visual, auditory and somatosensory signals to initiate motor commands. The pons relays sensory information to the medulla and cerebellum. The medulla oblongata controls autonomic activities, such as heartbeat and respiration and transmits sensory signals between the spinal cord and the higher parts of the brain. The reticular formation functions as a “first alert” system to arouse and react by means of the reticular activating system (RAS).

Orienting behaviors function as exploration and response to the environment at a distance by eyes, ears, and nose, as well as by receptors on the body surface. Orienting involves three components—perception, arousal, and movement. The orienting begins with sensory signaling input from olfaction, vision, hearing, and bodily sensory systems, all of which are integrated in the brainstem (Sprague, 1996). Many conventional theories of emotion still assume that all sensory input is transmitted to the cortex for processing. Yet, it has been known since the 1970’s that this assumption is not valid for critical aspects of orienting (Antonini et al., 1979). In multiple orienting experiments on cats, for instance, Polish neurophysiologist Boguslaw Zernicki reported that removing the superior colliculus completely eliminated the ocular-following REFLEX, while the REFLEX remained intact after bilateral ablation of the visual cortex, removal of the frontal oculomotor cortex and transection of the corpus callosum (Zernicki, 1988). How then does the orienting REFLEX lead to action without cortical input? Answering this question has led to intense interest in the development and function of the various nuclei within the brainstem, including the inferior and superior colliculus, the pons, the reticular formation and the medulla oblongata (Figure 4). The neural circuitry in these structures is now being studied more closely in the fields of physiology, behavior, development and evolution (Kelley et al., 2020).

The human brainstem structures are highly conserved across subphyla, having evolved in early fish, approximately 505 million years ago (McMullan and Pilowsky, 2010). Collectively, these structures are at the neuronal crossroads of all sensory processing and integration. They integrate cardiovascular system control, respiratory control, pain sensitivity control, alertness, awareness, even consciousness (Redgrave et al., 1987; Borday et al., 2004; Lavezzi et al., 2010).

Recent empirical findings suggest that the mechanisms supporting sensory integration are more complex than previously believed. Remarkably, the brainstem does not have the ability to integrate multi-modal sensory information at birth. Such integration typically develops in early life, as experience with cross-modal sensory cues is acquired. Disruptions in the way midbrain circuits acquire multisensory experience affect multisensory processing capabilities and lead to atypical neurodevelopmental trajectories, such as that seen in autism (Cuppini et al., 2018; Burstein and Geva, 2021).

The pontomedullary reticular formation in the brainstem is best known for its role in promoting arousal and consciousness (Khroud et al., 2021). This region is involved in regulating the sleep–wake cycle and filtering incoming stimuli to discriminate irrelevant background stimuli. Reticular formation neurons also form circuits with the motor nuclei of the cranial nerves; these nuclei contain neurons that are responsible for motor movements in the face and head, as well as motor movements related to autonomic functions of the visceral organs. The fibers that arise from these locations combine with other pathways that ascend to the cerebral cortex and thalamus to promote wakefulness, vigilance, and overall arousal. These pathways from the reticular formation must be functional for normal attentional abilities and sleep–wake cycles to be preserved.

The reticulospinal system is a distributed network of neurons extending from the caudal midbrain through the pons and medulla (Peterson, 1984). The fundamental architecture and role of the reticulospinal system persists across species: reticulospinal neurons are large cells with dense arborizations and large fast-conducting axons that descend in the spinal cord, forming synapses with interneurons and motoneurons in multiple segments to produce movement.

**Figure 5:**
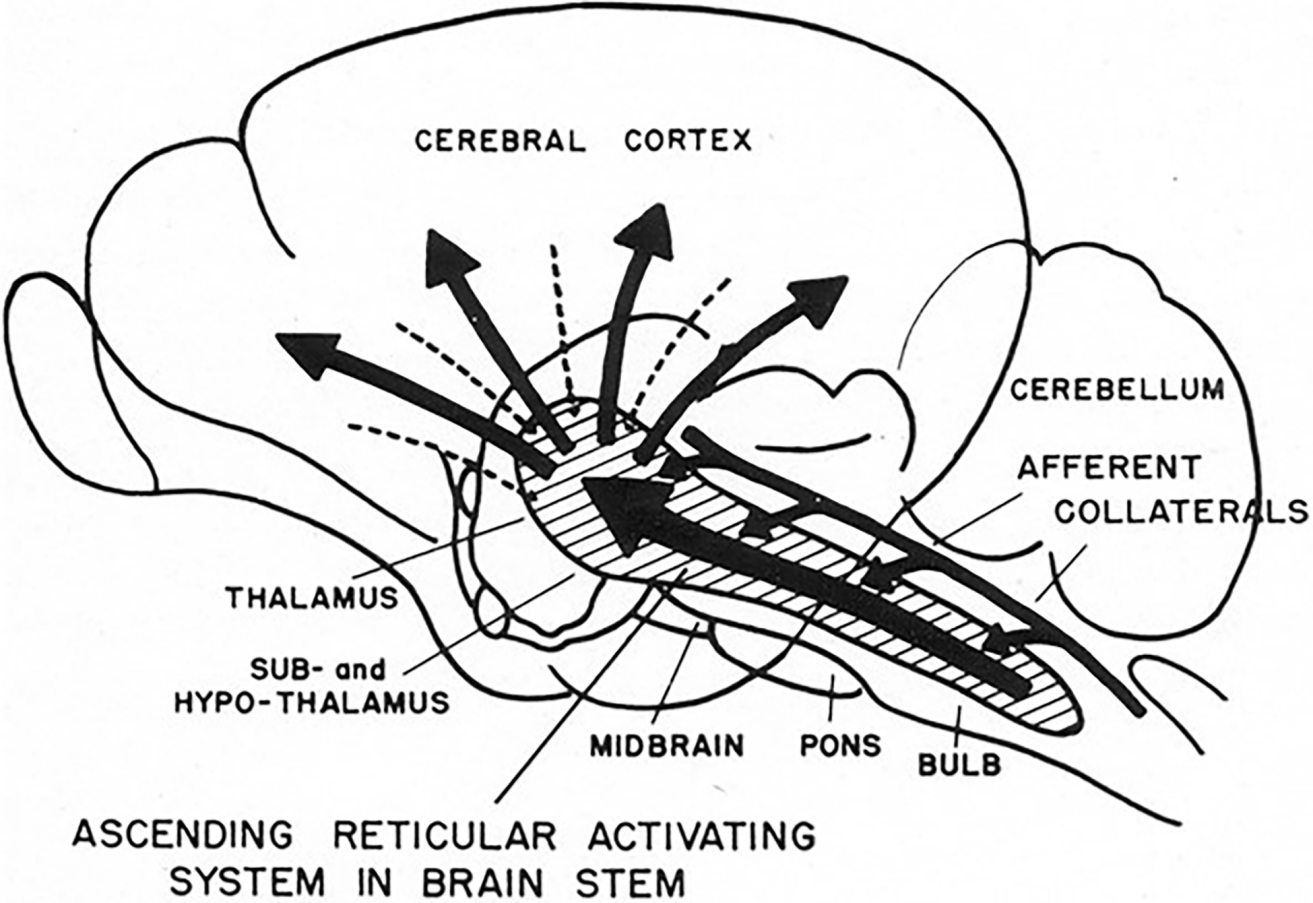
**1951 conceptual drawing of the ascending reticular activating system (ARAS)**. Note there is no indication of descending signals to the heart. (From Starzl et al., 1951) (Used with permission of the American Physiological Society).

It is now known that sensory signals entering the superior colliculus are simultaneously transmitted in two directions, *superiorly* to upper brain centers (thalamus, cerebellum, amygdala and cortex) and *inferiorly* to the viscera (heart, lungs) and spinal nerves *via* giant reticular neurons (Sprague, 1996). Thus, neurotransmitter signaling constitutes a first-alert communication network, reaching the entire body by way of the reticular activating system (RAS) (Figure 5).

Discovered in the mid-twentieth century (Wijdicks, 2019), the RAS functions as a kind of signaling highway (see Figure 5). A collection of distinct nuclei—more than 20 on each side in the brainstem—connect to the pons, medulla, as well as to the posterior hypothalamus (Nolte et al., 2009). These nuclei release neurotransmitters, such as dopamine, norepinephrine, serotonin, histamine, acetylcholine, glutamate and oxytocin in response to stimuli.

**Figure 6:**
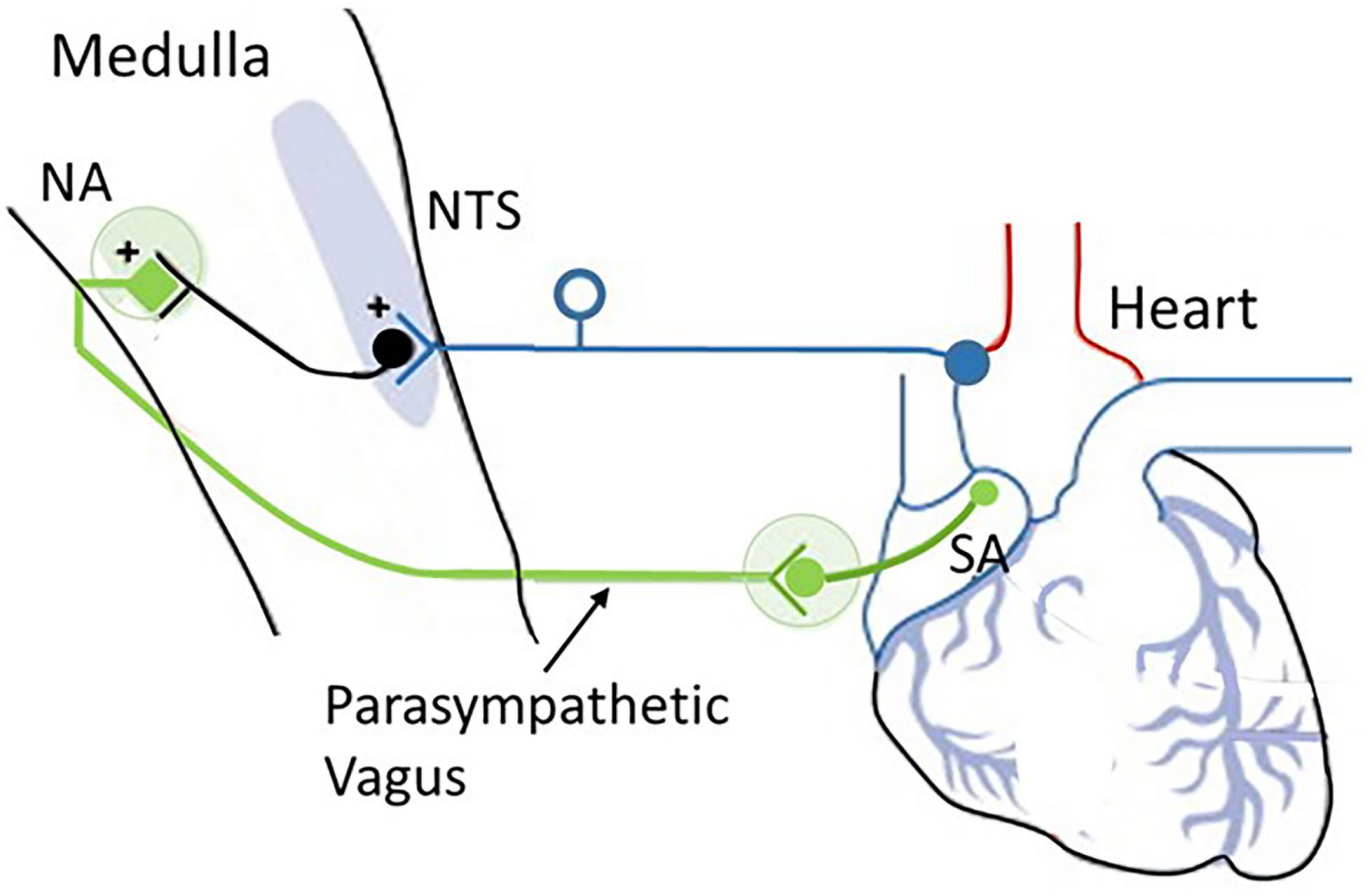
**The cardioinhibitory baroreflex** **(vagally mediated cardiac bradycardia)**. Baroreceptor afferents (dark blue) synapse at the nucleus of the tractus solitarius (NTS). The vagal component of the baroreflex (green) runs from the NTS to the nucleus ambiguus (NA) and sends efferents to the sinoatrial node (SA) to regulate heart rate. Source: Low and Tomalia (2015). Reproduced under the terms of Creative Commons Attribution Non-Commercial License (http://creativecommons.org/licenses/by-nc/3.0/).

Reticular formation circuitry helps coordinate the activity of neurons in the cranial nerve nuclei. Thus, the RAS is involved in the regulation of simple homeostatic reflex functions, such as blood pressure and heart rate. For example, reticular formation neurons in the medulla facilitate cardiac inhibition associated with the parasympathetic fibers of the vagus nerve (Figure 6). This activity also involves motor functions of the gastrointestinal system (e.g., swallowing, vomiting), respiratory functions (e.g., coughing, sneezing, breathing rhythm), and other cardiovascular functions (e.g., maintenance of blood pressure). Reticular neurons in the medulla and pons also contribute to orofacial motor responses by coordinating activity in motor nuclei for the trigeminal, facial, and hypoglossal nerves, including emotional facial expressions, like laughing or crying, as well as for coordinating eye movements.

The medulla integrates many vital survival reflexes, such as the defense and escape reflexes. In addition to those that control swallowing, vomiting, and coughing, the medulla oblongata is the primary site of cardiovascular and baroreflex integration. Sensory signals in the NTS transmit to vagal preganglionic parasympathetic neurons located in the ventrolateral portion of the NA and stimulate preganglionic efferent vagal fibers to the heart, resulting in bradycardia. Thus, the baroreflex regulates the heart by way of a simple autonomic reflex arc along sympathetic and parasympathetic pathways (Cutsforth-Gregory and Benarroch, 2017; see Figure 2).

The baroreflex-cardioinhibitory pathway involves a direct input from the NTS to cholinergic cardiac ganglion neurons that inhibit the automatism of the sinus node and elicit bradycardia (i.e., slowing of heart rate). In this region, the nucleus of the NTS serves as the primary site for the first synapse of the baroreceptor afferents and is the key integrating site for baroreceptor input including the cardiopulmonary baroreceptors. The NTS is a bilateral structure that receives monosynaptic inputs from afferents using glutamate as the primary neurotransmitter. The NTS integrates and relays baroreceptor afferent information *via* a polysynaptic pathway to other important medullary centers to control parasympathetic and sympathetic pathways to the heart and blood vessels.

That the cardiac orienting response is neurogenic is supported by, among other things, the time course (Porges, 1995). First, heart rate deceleration associated with the cardiac orienting reflex is rapid, occurring under 1 s, and under normal conditions returns to baseline rapidly. Second, the latency characteristics of the cardiac orienting REFLEX are like other neurogenic bradycardic reflexes, such as optovagal, vasovagal, baroreceptor-vagal, and chemo-receptor-vagal reflexes, which depend upon rapid activation.

An interesting and seemingly paradoxical fact regarding signal transmission time is that parasympathetic nerve traffic enacts its *inhibitory* effects at a much faster rate (less than 1 s), compared with the rate of sympathetic excitatory outflow (more than 5 s). Unlike the sympathetic innervation, which must first synapse within chain ganglia to supply the heart with postsynaptic fibers, the parasympathetic fibers synapse at ganglia located directly on the heart and short postsynaptic fibers that supply the target organ. While heart rate focuses on the average beats per minute, heart rate variability (HRV) measures the specific changes in time (or variability) between successive heart beats. The time between beats is measured in milliseconds (ms) and is called an “R-R interval” and is considered a reflection of vagal outflow (Nunan et al., 2010).

In summary, in terms of responding to novel signals in the environment recent brainstem signaling research suggests that evolution has favored the shorter “inhibitory” reflex over longer “excitatory” response time, suggesting it is time to reassess conventional theories of emotion.

### Autonomic Function and Behavior

**Figure 7:**
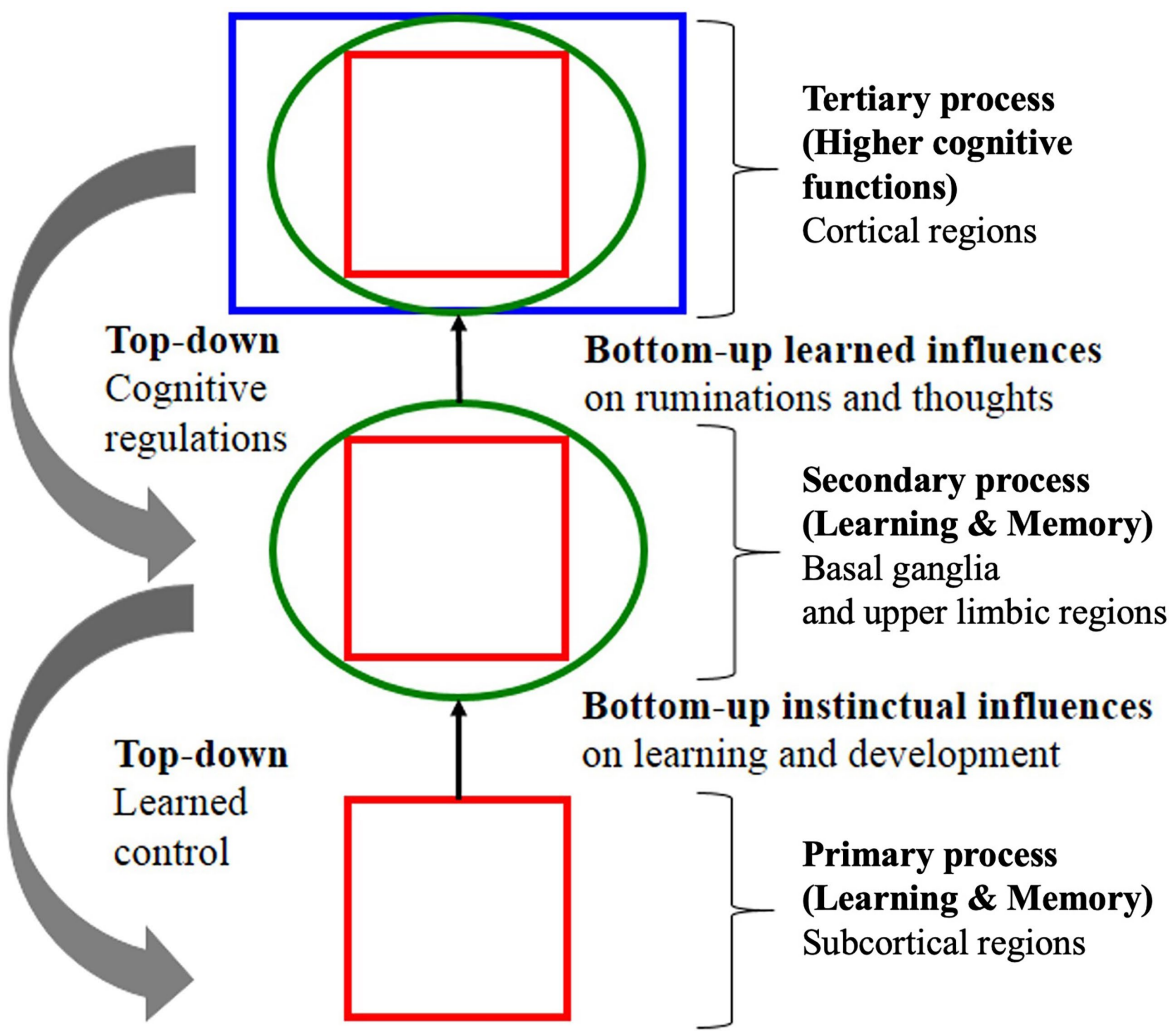
**Example of a theoretical signaling pathway showing hierarchical “top-down” self-regulation of emotions by cortical regulators**. Note in this model, signaling is entirely internal and environmental signaling is not accounted for. Note also the deprecatory relationship between primary “bottom-up” “instinctual influences” (**lower brain function**) and secondary and tertiary processes of the cognitive system (**higher brain function**) adapted with permission from Panksepp and Solms (2012).

Since Descartes, in both science and medicine, theorists have almost exclusively linked “*behavior*” to psychological function (Panksepp and Solms, 2012; Damasio and Carvalho, 2013). Contemporary theories variously define emotions as “instinctual influences,” “bodily impulses” or simply “reflexes” stating that “*emotions*” arise from autonomic function (Tyng et al., 2017; Figure 7). Such theories promote the idea that the way to control the autonomic emotional “REFLEX” is to change the psychological “RESPONSE.” The process by which the cortex exerts control over emotional impulses is often difficult to follow:

“*Primary emotional processing for homeostatic, sensory and emotional affects facilitate secondary learning and memory processing via a “SEEKING” system that promotes survival and reproductive success (bottom-up instinctual influences). As secondary processes are continually integrated with primary emotional processing, they mature to higher brain cognitive faculties to generate effective solutions for living and subsequently exert top-down regulatory control over behavior. The primary emotional processing is mediated by complex unconditioned emotional responses (evolutionary “memories”) through “Law of Affect”; sometimes called “reinforcement principle” that explains how the brain’s emotional networks control learning. This bi-circular causation for higher brain functionality is coordinated by lower brain functions*” (Tyng et al., 2017).

In the 1980’s, the idea that the cortex is separate from autonomic function was challenged by psychologist Bernard T. Engel, PhD (1928–2017). Engel proposed a radical idea, that autonomic functions related to *circulation* (i.e., heart rate, blood pressure, breathing, etc.) should themselves be considered behavior. He made a simple observation. Yoga practitioners in India arguably use a form of *operant conditioning* to consciously control autonomic reflexes (i.e., heart rate, blood pressure and breathing). If behavior is defined as that which is controlled psychologically, and autonomic function can be controlled psychologically, then autonomic function should be considered *behavior* (Engel, 1986). Despite his excellent review of the conditional reflex literature and the compelling logic of his argument, Engel’s work was never accepted. His work is today largely forgotten and has arguably had no appreciable impact on the field of psychology or medicine. As Engel’s contemporary researcher and another proponent of autonomic conditioning, W. Horsley Gantt, wrote in 1964, “*both psychology and medicine are reluctant to acknowledge that the conditional reflex extends to the autonomic system*” (Gantt, 1964).

### Arousal and Command

**Figure 8:**
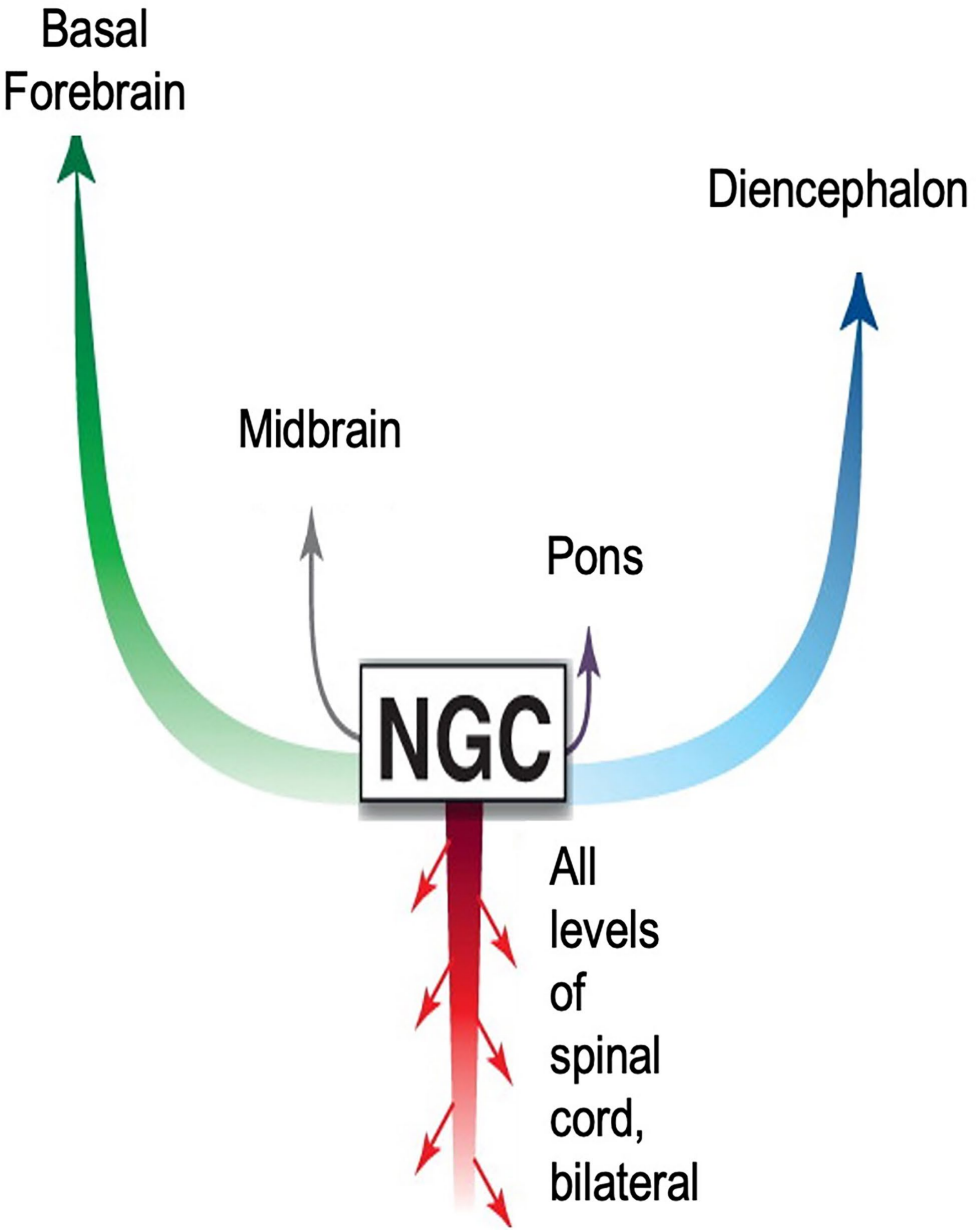
**Diagram accentuating importance of CNS signaling pathways from the nucleus reticularis gigantocellularis (NGC) to survival movements and behavior**. The NGC is located in the reticulospinal tract of the brainstem. It consists of giant pairs of monosynaptic command neurons that respond to glutamatergic stimuli. The NGC form a “first response” network that transmits novel information from the environment to all parts of the body. The network is very similar in form and function to those found in reptiles and fish. As with nearly all theoretical models describing signaling pathways regulating emotions, this model emphasizes ascending pathways to higher brain centers. Descending pathways are limited to the spinal cord. Missing entirely is the ANS neurohormonal pathway to the entire body *via* cardiovascular circulation. Adapted with permission from Pfaff et al. (2012).

The boundaries of the nuclei within the brainstem are somewhat imprecise. This fact, combined with disparate neuronal types within each nucleus, has led to some inconsistencies in nomenclature, and to difficulties in understanding reticular formation control of movement (Brownstone and Chopek, 2018). However, the consensus is that behavioral commands, conscious and unconscious begin in the reticular formation and are transmitted to the CNS *via* pairs of giant monosynaptic command neurons, collectively referred to as nucleus reticularis gigantocellularis (NGC; Figure 8). Although known, conspicuously absent from this anatomical description is the ANS pathway *via* the heart and the role neurohormones have on body-wide *state*.

Until recently, physiologists assumed escape behaviors mediated by the reticulospinal system are fixed and stereotypical. However, recent findings indicate the behaviors are variable, adaptable and *state dependent* (Liu and Hale, 2017).

In 2006, functional neuroscientist Donald W. Pfaff (1939) proposed a construct, *generalized arousal*, to describe the underlying neuronal system that needs to exist to produce successful sexual behavior in the adult vertebrate (Pfaff, 2006). Building on the established knowledge base on reticulospinal system and associated behaviors, Pfaff proposed that a successful generalized arousal system must be alert to all sensory stimuli, capable of producing voluntary motor activity and possess emotional reactivity (Pfaff et al., 2012). In addition, a generalized arousal system:

- Must be labile, not sluggish and *sensitive to the momentary state of the organism*.
- Must converge all sensory stimuli to activate and support the same set of arousal subsystems.
- Must diverge to activate cerebral cortex, autonomic nervous systems, and endocrine organs to initiate behavior.
- Must be robust and cannot fail. The survival of the organism depends on adequate CNS sexual arousal.

### State vs. Trait

Traditionally, psychology and psychiatry have defined behavioral disorders according to characteristic cognitive, personality, behavioral, and psychiatric phenotypical patterns or *traits*.

**Figure 9:**
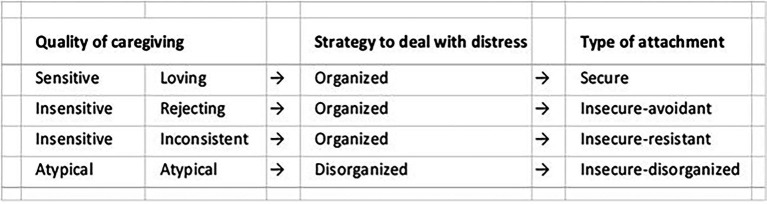
**Attachment Types**. According to Bowlby’s attachment theory, infant behavior is self-regulated by conscious brain mechanisms. Behavioral disorders are identified according to four attachment types or style.

John Bowlby’s psychologically based attachment theory (Bowlby, 1969) is widely accepted and cited to support interventions treating infant and child socioemotional disorders. Behavioral disorders are characterized according to four psychological attachment *styles* (i.e., traits; Figure 9). For over 60 years, attempts have been made to correlate attachment styles with genetics, brain mechanisms, oxytocin, cortisol and other physiological measures, without success (Benoit, 2004). Findings often do not apply equally to all patterns of attachment, to all ages, or all temperament styles, among other factors. In addition, some important evidence is indirect, coming from studies that investigate the impact of variations in caregiving quality, such as differences in maltreatment and separation from caregiving figures, and on biology, and vice versa, rather than looking at differences in attachment *per se*, which complicates their interpretation.

In the late twentieth and early twenty-first centuries, multiple conceptual models were proposed to link neurological theory with behavioral theory by means of a functional cerebral systems framework (Porges, 1997; Geva and Feldman, 2008). A consensus emerged that frontal regions exert regulatory control over posterior systems for autonomic function and behavior in a dense, interconnected network. Impairment at levels within the system was found to influence cognitive processes and behavior depending on the extent of frontal regulatory capacity.

Interestingly, Engel’s point that *circulation is behavior* seems to have gained support from recent research on the neural underpinnings of psychological function that suggests some behavior might be the result of physiological *state*, as opposed to a longstanding psychological *trait*. For instance, converging evidence from multiple fields supports the idea that orienting conflict or “anxiety” behaviors are not *traits*, but are driven by *autonomic state*. Research now suggests psychological function is driven by an underlying relationship of the two opposing electro-physiological state actions, excitation and inhibition (E/I). Importantly, findings suggest certain *behavioral phenotypes or traits* may be correlated with E/I balance or imbalance (Liu et al., 2021).

### Approach-Avoidance

Among the longest standing and puzzling behaviors associated with the orienting phenomenon are approach-avoidance behaviors. The body must constantly adjust levels of arousal and attention to different kinds of sensory stimuli. How do modulatory systems synergize to shape global brain states, which influence the way the cortex processes sensory information and drives behavior?

**Figure 10:**
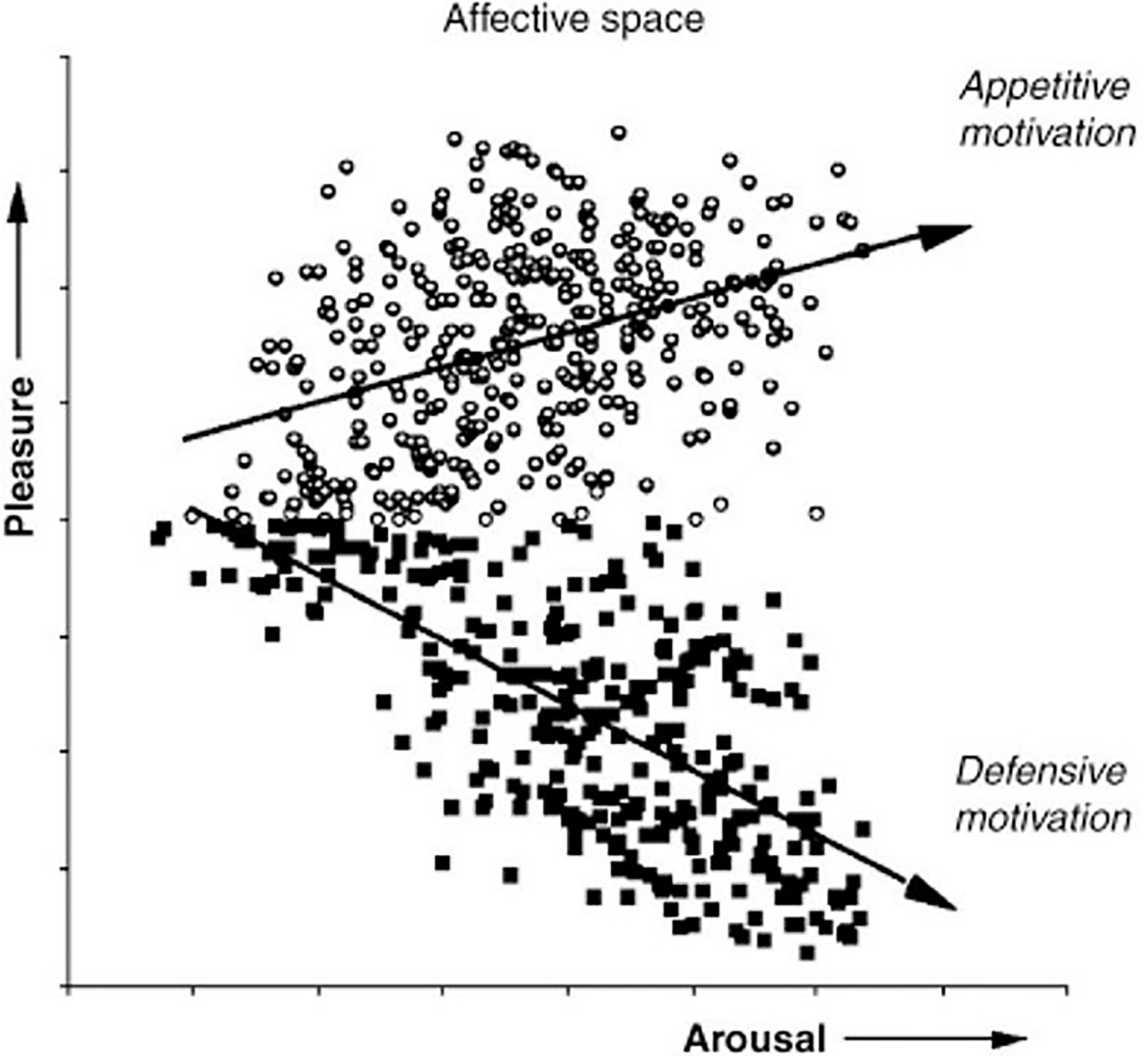
**Appetitive vs defensive orienting motivation and emotion**. The ratings of pleasure and arousal are consistent with the hypothesis that evaluation of evoked response potential in the frontal cortex reflects the level of subcortical activation in conserved autonomic appetitive or defensive motivational systems. (From Bradley, 2009, permission John Wiley & Sons, Inc.)

Studies by Mary M. Bradley and Peter J. Lang (1930) at the University of Florida’s Center for the Study of Emotion and Attention test the theory that orienting activates evolutionarily conserved defensive and appetitive motivational systems that evolved to protect and sustain the life of the individual (Lang et al., 1993). Figure 10 plots the results of a 1993 study that measured electrical evoked response potential (ERP) in the frontal and parietal cortex, autonomic physiology and behaviors during picture viewing. The experiment confirmed the hypothesis that emotional approach or avoidant behaviors are organized along two strategic dimensions of affective valence and arousal, which represent primitive motivational parameters integrated in *subcortical brain centers* (Bradley, 2009).

The prefrontal cortex is essential for cognitive flexibility and decision-making. These functions are related to reward- and aversion-based learning, which ultimately drive behavior (Jones and Graff-Radford, 2021). Evidence suggests that bottom-up amygdaloidal neuromodulation of the prefrontal cortex by neurotransmitters such as dopamine, acetylcholine and serotonin is essential for the top-down control of reward- and aversion-based behavior (Pastor and Medina, 2021). The amygdala is currently theorized to be a brain hub for integration of sensory and nociceptive information (Janak and Tye, 2015). However, studies using new technology suggest that prefrontal-amygdaloidal responses are themselves driven by modulatory microcircuit mechanisms in the brainstem.

The prefrontal cortex is composed of several neuronal subtypes. Broadly, they can be classified as excitatory pyramidal neurons (glutamatergic) and inhibitory interneurons (GABAergic; Somogyi et al., 1998). The microneuronal balance between excitation and inhibition is crucial for proper cognitive processing. For instance, the cellular balance of excitation and inhibition (E/I balance) within neural microcircuitry has been linked to severe behavioral deficits in psychiatric diseases, such as autism and schizophrenia (Rubenstein and Merzenich, 2003). Dysregulation of these systems can modify neural activity across the forebrain, and thereby affect the progressive refinement and emergent efficiencies of forebrain-processing systems.

The neural substrates of approach-avoidance behaviors are also being probed by way of excitatory/inhibitory (E/I) state balance. New techniques that combine circuit, genetic, and imaging are helping to understand the modulatory coding of various neurotransmitters and their overlaps/dissociations. For instance, Yizhar et al. used several novel optogenetic techniques to investigate the causes of cellular E/I balance in the microcircuit physiology of freely moving mammals. They found that elevation, but not reduction, of cellular E/I balance within the mouse medial prefrontal cortex elicited behaviors consistent with severe neuropsychiatric symptoms observed in humans (Yizhar et al., 2011).

Prefrontal cortical neurons maintain E/I balance through the release of neurotransmitters, such as dopamine, norepinephrine, serotonin, histamine, acetylcholine, glutamate and oxytocin. As reviewed above, reticular formation circuitry in the brainstem helps process and coordinate novel sensory signaling activity by way of giant command neurons, which regulate reflex functions, such as blood pressure and heart rate through the release of many of the same neurotransmitters. Since the reticular activating system serves as a first alert system, it is reasonable to assume that signaling from the brainstem, in addition to modulating the rest of the body, modulates E/I balance in the brain, including the amygdala and prefrontal cortex. In other words, evidence now suggests that approach-avoidant behaviors may be *state-driven*, as opposed to *trait-dependent*.

Most recently, the COVID-19 pandemic has presented a singular opportunity to illustrate how research in cell biology, biochemistry, immunology, and cytochemistry links disruptions in socioemotional communication to immune system disruptions. Pandemic-associated social restrictions have revealed the loss of an essential stress buffer and important parameter for general mental and physical health: *social support* (Gryksa and Neumann, 2022). Chronic social isolation and lack of social support are impacting not only mental health, but also the body-wide oxytocin system and the immune system.

As reviewed above, neurohormones (chemical messenger molecules) are released by neurons in the brainstem and enter the bloodstream, where they travel to distant target sites throughout the body. Two well-known examples of neurohormones associated with socioemotional behavior are oxytocin and vasopressin. As the two systems are closely linked to stress regulation, the activation of the two hormone systems even by subtle social stimuli is of particular relevance in the context of the two hormones as a mediator of the positive or negative effects of social contact on stress responsiveness. For instance, oxytocin and vasopressin exert opposite regulatory effects on cellular homeostasis, including mitochondria and reactive oxygen species, which are closely related to cellular inflammatory responses. While oxytocin is known to inhibit inflammatory pathways like oxidative stress and protein translation abilities during cellular stress, vasopressin amplifies inflammatory responses (Welch and Klein, 2009; Klein et al., 2016; Bordt et al., 2019).

Together, these findings support the hypothesis that socioemotional body-wide state changes at the behavioral as well as physiological level are linked to changes at the cellular and circuit level. They also lend support for Engel’s contention that the movement of molecules in the blood through the vessels of the body induced by the pumping action of the heart should be viewed as behavior.

### Historical Review Summary

We have briefly reviewed approximately 500 years of scientific literature on the human nervous systems and the reflex arc concept. We have seen the definition change over time. Established reflex arc theory and ideas changed as new evidence accumulated that pointed to a new understanding. New theories emerged to align with the new evidence. Nonetheless, the assumption that internal conscious thought controls emotional behavior and associated physiology continues to drive nearly all research on infant behavior.

Scientific research, especially recent genetic research, is casting Descartes’ human mind-centric declaration in new light. As little as 1.5%–7% of the modern human genome is uniquely human. New genetic research suggests that most of changes specific to modern humans involved genes related to brain development and function and occurred only within the past 600,000 years (6). In addition, growing research suggests that the biological mechanisms controlling mother/offspring social behaviors evolved over hundreds of millions of years (Wilson, 2006; Strassmann et al., 2011; Wilson and Nowak, 2014). Nonetheless, science continues to proceed under the assumption that cortical structures, or that psychopharmaceutical drugs that target cortical structures, manage human emotional behavior on their own. The sheer volume of scientific data supporting this notion is immense and compelling. But, equally compelling and important are the exceptions and anomalies in the data that call for a reexamination of theory supporting the current paradigm. One thing is clear, however. The new biological evidence on socioemotional behavior is pointing to the need for a fundamental rethinking of assumptions.

## The Autonomic Socioemotional Reflex Arc

### On Instinct, Prematurity, and the Orienting Reflex

For all intents and purposes, the highly controlled modern NICU presents a laboratory for examining mother and infant behavior. Therefore, we will compare the mother and preterm infant with mother and full term infant to illustrate our theories on co-learning, instinctive behavior and the orienting reflex.

Among mammals, the period of gestation varies. However, as a rule, a baby born prematurely in the wild will not survive. At the same time, as a rule, if a mother is not attracted to the baby or avoids the baby, the baby will not survive (Numan et al., 2006). Humans are the singular exception to these rules. Humans alone have devised medical technology and procedures that enable infants born prematurely to survive at earlier and earlier gestational age and to develop, even despite occasions of avoidance by the mother. This unnatural premature birthing environment has produced unintended dire consequences. As a rule, compared with babies born at term age, prematurely born babies are at much higher risk for a wide range of life-long socioemotional, behavioral and developmental disorders than are those infants born at term age (Fitzallen et al., 2020). Nonetheless, there are the exceptions. Many infants develop relatively normally. Why do some preterm babies do well, while other babies struggle?

We have proposed that instinctive behaviors between mother and infant observed after birth are not inherited but are the result of autonomic co-conditioning during gestation (Ludwig and Welch, 2020). Given a normal, healthy term birth, gestational autonomic co-conditioning results in mother/infant *approach* orienting behaviors. A premature birth results in mother-infant separations and reactions that can lead to avoidance behaviors on the part of the infant, the mother or both the infant and the mother. Thus, mother/infant orienting behaviors fall somewhere between two opposite reactions— *approach* and *avoidance*. These two opposite behavioral reactions correspond to two physiological states. In one, the physiology of the mother and infant are in a state of co-regulation. In the other, the physiologies of the infant and mother are in a state of dysregulation. When the mother-infant physiologies are co-regulated, the two display approach behaviors. When the mother-infant physiologies are dysregulated, one or both display avoidance behaviors. This theoretical position leads to a simple syllogism:

- A baby’s socioemotional behavior correlates with the mother’s socioemotional behavior
- Socioemotional behavior correlates with autonomic state
- A baby’s autonomic state correlates with the mother’s autonomic state

How are the baby’s and mother’s autonomic states connected? To answer this question, we consider the orienting reflex arcs of a mother and a baby when they first experience one another after birth. We consider two conditions, one we will call *adaptive* and the other *maladaptive*, where adaptive results in prolonged approach behaviors and maladaptive results in prolonged or intermittent avoidance behaviors.

A newborn infant is physically connected to the mother or family member on more or less a continuous basis for the first weeks and months of life. Therefore, infants experience familiar sensory stimuli of the mother. The orienting between mother and infant in this condition we will call *adaptive*. In the medical treatment of infants born prematurely, however, infants often experience long periods of physical separation from the mother and family members. In such cases, at least in part due to physical and emotional separation, the natural mechanisms of the birthing process are interrupted, and the sensory stimuli of the mother and the infant can become unfamiliar to one another. Sometimes orienting behaviors between the mother and prematurely born infant are *maladaptive*. Using these two hypothetical adaptive and maladaptive conditions, we will speculate on how the multi-sensory stimuli between the mother and infant might impact the orienting reflex arc in the mother and infant and lead to two different set of behaviors.

In both the adaptive and the maladaptive conditions, the orienting reflex is triggered when a mother holds her baby face to face and generates sensory stimuli. As reviewed above, the sensory stimuli from both conditions are “integrated” in the brainstems of the mother and the infant. Integration is conventionally interpreted to mean that sensory signals are “integrated” in the brainstem and sent *via* ascending pathways to thalamo-cortico-amygdaloid structures for *processing* and *executive command*. However, this conventional interpretation is challenged when we consider what is currently known about the reticular activating system (RAS).

**Figure 11:**
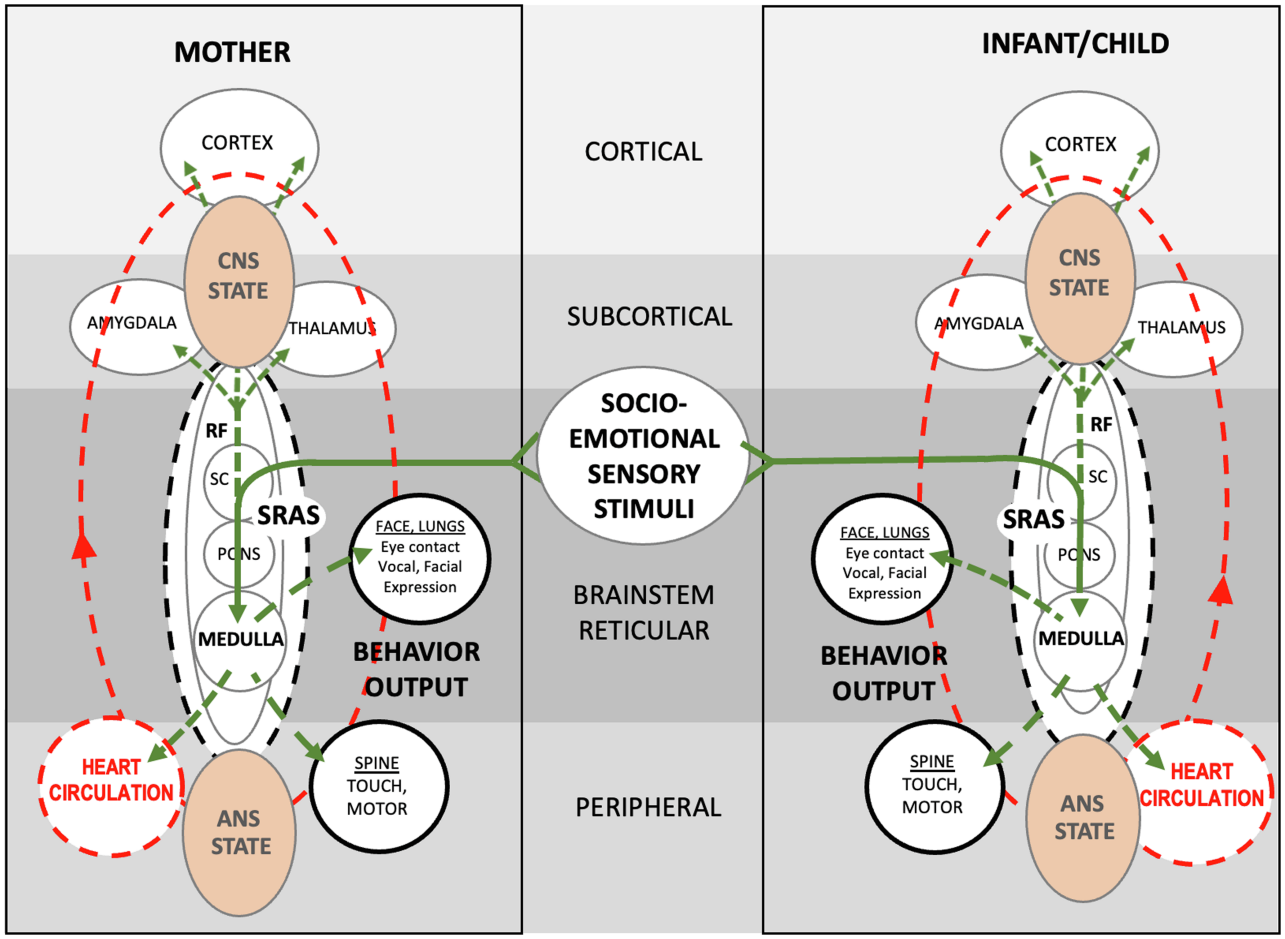
**Schematic showing theoretical autonomic socioemotional reflex (ASR) signaling arc**. The arc begins when multimodal sensory stimuli from close mother and infant proximity and face-to-face communication (**Solid green lines**) activate neuronal signaling (**Dotted green lines**) and cardiovascular signaling (**Dotted red lines**). Sensory stimuli integrate within the reticular formation, where neurotransmitters spark electrochemical/hormonal signaling based on prior autonomic conditioning. The signaling ignites the *systemic* reticular activating system (SRAS), which transmits more or less instantaneously throughout the body and brain *via* neuronal and circulatory pathways and producing a body-wide *autonomic state*. Note that signal transmission occurs in two ways; *via* *cardiovascular signaling* (circulation) from the medulla to the heart, and *via* *neuronal signaling* from the medulla to the spine and brain. The autonomic states of mother and infant determine *approach* or *avoidant* behavior. The entire autonomic socioemotional reflex (ASR) signaling arc, from sensory stimuli to behavioral output (**listed in solid black circles**), takes less than a second to complete.

Due to its critical role in survival and reproduction, the RAS is highly conserved across phyla and its structure and function differ little between fish and humans. Textbooks typically focus on the *ascending* (labeled ARAS) pathways that lead from the superior colliculus, pons, medulla and reticular formation to the cortex, based presumably on the fact that the RAS is required for normal, conscious functioning of the cerebrum (Figure 11; Nolte et al., 2009). Based on recent research on brainstem mechanisms, however, we propose a different explanation for sensory integration during the orienting REFLEX.

One characteristic common to all organisms is the dynamic ability to adjust internal physiology in response to environmental changes. The function of communicating with the environment is achieved through pathways that receive and process signals from the external environment. Individual pathways transmit signals along linear tracts resulting in regulation of discrete cell functions. This type of information transfer is an important part of the cellular repertoire of regulatory mechanisms (Jordan et al., 2000). Neurons communicate *via* both electrical signals and chemical signals.

Neurochemical cell signaling is well-described. Neurotransmitters can be small molecules or larger neuropeptides. The synthesis of small molecule neurotransmitters occurs locally - within the axon terminal, whereas neuropeptides, being much larger, and are synthesized within the cell body. These signals can hyperpolarize or depolarize their target neurons and thus neurons are excitatory or inhibitory based on the type of signal (Lodish et al., 2000).

Circulatory system and humoral signaling is a highly complex well-organized system, in which signal transduction plays critical physiological and pathophysiological roles. The cellular elements of the heart and vascular wall are equipped with an array of specific receptors and complex intracellular machinery that facilitate and drive appropriate responses to extracellular stimuli. The complexity is important because it allows cells to act in concert to maintain homeostasis by responding rapidly to small and fluctuating changes in the incoming environmental signals, while the crosstalk between signaling pathways allows coordinated responses to multiple different and sometimes opposing signals (Wheeler-Jones, 2005).

The recent review by Agirman et al. of cellular and molecular research over the past 5 years shows in great detail how the body responds to both internal and external signaling through circulation (Agirman et al., 2021). Also shown is how critical the gastrointestinal tract and its enteric nervous system are for detecting, integrating, and relaying external as well as internal sensory signaling (Welch et al., 2009; Klein et al., 2017). Immune cells throughout the brain and body continually survey environmental factors, eliciting responses that determine the brain’s psychological state and body’s autonomic state at any given moment. For instance, bidirectional communication of pro-inflammatory signals through the gut-brain axis is important for the regulation of physiological behaviors. The authors end their review on a telling note: “*How the* *brain regulates* [emphasis added] *intestinal inflammation in the efferent direction remains unclear*.”

In the case of a mother and baby, we propose a control mechanism separate from the thalamo-cortico-amygdaloid pathway. We posit that the socioemotional orienting between a mother and infant triggers the *systemic* reticular activating system (SRAS), with associated *neurochemical* and *circulatory signaling* that produces instantaneous body-wide physiological *states* in the pair. In our model, orienting stimuli trigger the release of neurotransmitters in the reticular formation that prime both signaling pathways.

Projections from the SRAS are important in determining whether the orienting stimuli results in approach or in avoidant behavior. The SRAS determines the socioemotional reaction between mother and baby upon sensory contact. Neurotransmitters, such as dopamine, norepinephrine, serotonin, histamine, acetylcholine, glutamate, GABA, and oxytocin are released from over 20 nuclei along the length of the brainstem into the giant reticular cells (NGC) and transmitted *via* ascending and descending pathways throughout the body (Nolte et al., 2009).

A complex interactive system of cellular and molecular signaling reflexes regulate or mediate cellular function, which in turn, mediate the behavioral reflex. The mix of neurotransmitters infused into the reticular formation determines whether the giant NGC neurons are charged with excitatory/pro-inflammatory or inhibitory/anti-inflammatory signals. The signals then transmit throughout the body *via* the neurochemical and neurohormonal circulatory pathways in less than a second, producing a momentary *autonomic state* and associated *approach* or *avoidant* behavioral REFLEX.

The key to understanding how socioemotional stimuli result in predictable behaviors lies in understanding how the signaling affects autonomic state in both the neuronal and circulatory pathways. For instance, an imbalance in expression of pro−/anti-inflammatory cytokines in the blood are now thought to be crucial players in immune dysfunction seen in ASD subjects (Nadeem et al., 2021). Another study associated disconnectivity of a brain functional network with blood inflammatory markers in depression (Aruldass et al., 2021). We hold that circulatory imbalances such as these and excitatory/inhibitory brain imbalances as reviewed above are all physiological correlates of mother/infant behaviors during orienting.

The vagal signaling pathway from the medulla to the heart is well described by psycho-physiologists with regard to orienting behaviors and social engagement (Porges, 1995; see Figure 2). The medullary mechanisms controlling the cardioinhibitory baroreflex (vagally mediated cardiac bradycardia) are well characterized as they relate to neurological pathologies (Cutsforth-Gregory and Benarroch, 2017; see Figure 6). Based on recent basic research findings and our clinical findings, we believe it is more likely that brainstem mechanisms regulate heart and circulation reflexively during orienting. In our example of mother/infant socioemotional engagement, sensory stimuli trigger systemic reaction *via* the SRAS, which releases pro- and anti-inflammatory cytokines into the circulation. Thus, circulating cytokines trigger systemic state-change, both autonomic and psychological. State-change in turn leads to two contrasting motor responses: approach or avoidance. As with other species, this highly conserved orienting reflex happens within a split second.

### Correlation of “Binary” Behavior and “Binary” Physiology

**Figure 12:**
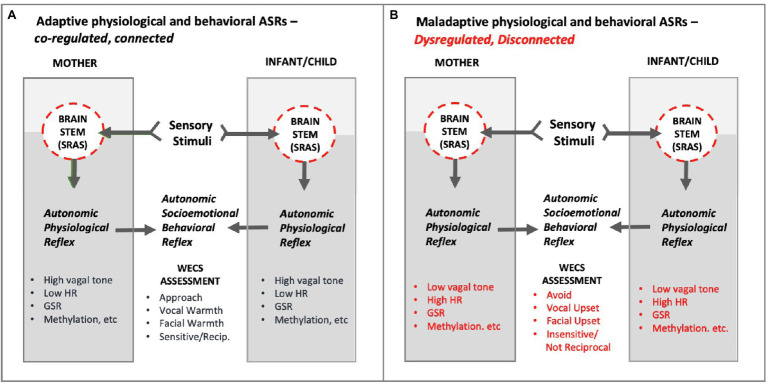
**Conceptual diagram of hypothetical Adaptive and Maladaptive Autonomic Socioemotional Reflexes (ASRs) in mother and infant/child**. As shown, the ASRs manifest behaviorally and physiologically. The mutual behavioral reflex component can be measured via the WECS. The mutual physiological reflex component can be measured by vagal tone, heart rate (HR), galvanic skin response (GSR), methylation, E/I balance, inflammatory/anti-inflammatory, etc. **Panel A** illustrates adaptive *autonomic socioemotional reflexes*. **Panel B** illustrates maladaptive *autonomic socioemotional reflexes*. The two conditional states correspond to long observed binary behaviors, such as approach/avoidance, and corresponding reports of binary physiology associated with behavior, such as those listed above. Abbrev: SRAS, systemic reticular activating system; WECS, Welch Emotional Connection Screen; HR, heart rate; GSR, galvanic skin response; E/I balance, excitatory-inhibitory balance.

Our ASR theory of emotions predicts a correlation between binary behavioral observations, such as “approach/avoidance,” and binary neuronal signaling, such as “excitatory/inhibitory.” Several important insights stem from this position (Figure 12):

- Autonomic state is fluid and constantly changing. Depending on environmental conditioning (e.g., mother/infant sensory stimuli), the ASR can be adaptive or maladaptive. We argue that so-called maladaptive mother/infant behavioral “traits” are behaviors temporarily “stuck” in a conditional excitatory “state.” Modifying the environmental conditions (e.g., the sensory stimuli between mother and infant) can rapidly modify autonomic state. A mother and her prematurely born infant are evolutionarily “wired” to modulate (calm) one another’s autonomic state upon physical contact. However, a change in environmental conditions (separation due to hospitalization) can change the autonomic socioemotional signaling from approach to avoidance and the autonomic physiological signaling upon contact from inhibition to excitation in one or both of them.
- According to ASR theory, mother/infant behavioral “traits,” whether adaptive or maladaptive following birth, are the result of autonomic conditioning. It is therefore theoretically possible to modify behavioral traits through autonomic conditioning. An example of such conditioning is Family Nurture Intervention (Welch et al., 2012), which Welch designed to modify and shape the ASRs between mother and preterm infant while in the hospital following birth.
- The *Welch Emotional Connection Screen* (*WECS*; Hane et al., 2019) is a behavioral assessment tool built on ASR theory. The WECS measures four modes of parent and infant/child ASR signaling behaviors at any given moment of close physical contact. Each of the four modes of ASR behaviors fall on a graded spectrum between two extreme behavioral constructs labeled *emotionally connected* and *emotionally not connected*. The significance of the WECS is that it is the first assessment tool to categorize *mutual* socioemotional behaviors, as opposed to *individual* socioemotional behaviors.
- Each of the modes of mother/infant behaviors measured on the WECS (e.g., mutual attraction, mutual vocal communication, mutual facial communication, sensitivity/reciprocity) theoretically correlate with concomitant binary ASR autonomic physiology (e.g., heart rate, breathing, galvanic skin response). In this sense, the WECS provides a lens on the autonomic effects of the parent–child relationship, which is an important predictor of future infant/child socioemotional behavior and relationships.

### Wired to Connect

Embedded in our theoretical position is the idea that at birth the mother and infant have in place the neuronal wiring and neurohormonal and neurotransmitter reflex mechanisms that enable them to connect to one another at the level of their *autonomic nervous systems*, without the benefit of cortical processing (Figure 12). We have demonstrated the importance of this autonomic connection in our clinical trials among premature infants (Welch et al., 2020). Over the past 15 years, research from multiple fields supports a view of mother/infant socioemotional behavior that is radically different from the current paradigm. The brainstem and heart, not just the brain, are at the center of that view.

Relevant to this last point, we quote the abstract from psychologist Bernard Engel’s 1986 paper, *An Essay on Circulation as Behavior* (Engel, 1986).

“*Most conceptual models of the organization of the cardiovascular system begin with the premise that the nervous system self-regulates the metabolic and non-metabolic reflex adjustments of the circulation. These models assume that all the neurally mediated responses of the circulation are reactive,* i.e.*, reflexes elicited by adequate stimuli. Responses of the circulation are conditional in three senses. First, as Sherrington argued, reflexes are conditional in that they never operate in a vacuum but in a context together with other reflexes. Guided by functional utility, they interact rather than add. Second, as Pavlov argued, stimuli acquire meanings as a result of experience. This notion of stimulus effect plus Sherrington’s notion of conditionality suggests that association is one of the ways stimuli eliciting cardiovascular reflexes acquire their meanings and thus their relative strengths. Finally, as Skinner and others have argued, operants are responses that act upon the environment to obtain consequences - that is, stimuli. As operants, cardiovascular responses fulfill a major biological need, functioning proactively. The cardiovascular response is an integral component of the animal’s behavior regardless of whether it is an elicited reflex or the eliciting stimulus acquired its properties as a result of the genetic inheritance of the animal or through experience, or the cardiovascular response is emitted in anticipation of an environmental consequence*” (Engel, 1986).

### Significance

Collectively, these theoretical insights provide a new framework with which to view and assess the mutual *autonomic health* of the mother and infant/child relationship, as opposed to the mental health of the individual. Viewing the *mutual* behaviors of mother and infant in terms of their ASRs offers a simple way to assess autonomic relationship and identify those pairs that need help.

The WECS ASR assessment tool was validated with conventional instruments and correlated with autonomic function (Hane et al., 2019). It was also validated in full term preschool-aged children (Fagan et al., 2019; Frosch et al., 2019; O’Banion et al., 2021). These findings support the theory presented here.

Viewing mother/infant socioemotional relational behaviors in terms of a state-driven autonomic conditional reflex opens possibilities for changing the relationship from maladaptive to adaptive through autonomic counter-conditioning methods, such as the FNI calming cycle (Welch, 2016). The theoretical advance of the WECS is that it can serve as a measure of relational health between mother and infant/child to determine when and how to intervene. Additionally, ASR theory opens up new possibilities for early parent–child intervention, including proactive family behavior as prevention. In terms of research, ASR theory and the WECS open up an entirely new category of testable scientific hypotheses that correlate behavioral phenomena with physiology.

### Limitations

The ASR signaling pathway remains theoretical and awaits further testing. Also, correlation between behavior and physiology does not prove causation. Of course, the same limitation should apply to conventional cortical signaling pathway theories, which have for too long been supported by *post hoc* research findings.

## Summary

The ASR theory presented here challenges very long-held scientific assumptions about emotional behavior. Conventional theories hold that individual cortical RESPONSE mechanisms control mother and infant socioemotional behaviors, and that they are best changed or modified using operant conditioning. We propose that mutual autonomic REFLEX mechanisms control mother/infant socioemotional behavior, and that they are best changed by using subconscious autonomic conditioning (Ludwig and Welch, 2020). Conventional theories state that behavior is individual and self-regulated. We propose adaptive mother/infant behaviors are mutual and co-regulated, and that mother/infant autonomic states are relational and contingent.

There are few core beliefs more engrained in conventional Western science than the idea that the individual consciously chooses among alternatives and controls behavior, independent of natural, social (or divine) restraints. Yet, new research data is pointing to a new reality. The skeptic says that *seeing is believing*, but we must always be mindful of the fact that believing sometimes hinders the scientist’s ability to see.

## Author Contributions

RL conceived the design of the review, conducted the literature review, drafted the manuscript, including intellectual content, investigated and resolved all theoretical issues related to the work. MW contributed the foundational insights for this work from her medical training, clinical practice, and her basic and clinical research, revised the manuscript critically for important intellectual content. Both authors approved the final version for publication, and are accountable for all aspects of the work related to the accuracy or integrity of the work.

## Funding

This manuscript was supported through generous gifts from the Einhorn Family Charitable Trust, the Einhorn Collaborative, Fleur Fairman, Mary Stephenson, The Donald and Mary Catherine Huffines Family, Kathy Emmett and David Golub. The funders were not involved in the study design, collection, analysis, interpretation of data, the writing of this article or the decision to submit it for publication.

## Conflict of Interest

The authors declare that the research was conducted in the absence of any commercial or financial relationships that could be construed as a potential conflict of interest.

## Publisher’s Note

All claims expressed in this article are solely those of the authors and do not necessarily represent those of their affiliated organizations, or those of the publisher, the editors and the reviewers. Any product that may be evaluated in this article, or claim that may be made by its manufacturer, is not guaranteed or endorsed by the publisher.
